# WNT11-Conditioned Medium Promotes Angiogenesis through the Activation of Non-Canonical WNT-PKC-JNK Signaling Pathway

**DOI:** 10.3390/genes11111277

**Published:** 2020-10-29

**Authors:** Jingcai Wang, Min Gong, Shi Zuo, Jie Xu, Chris Paul, Hongxia Li, Min Liu, Yi-Gang Wang, Muhammad Ashraf, Meifeng Xu

**Affiliations:** Department of Pathology and Laboratory Medicine, University of Cincinnati Medical Center, Cincinnati, OH 45267, USA; Jingcai.Wang@nationwidechildrens.org (J.W.); Gongmin0794@suda.edu.cn (M.G.); drzuoshi@gmc.edu.cn (S.Z.); jie.xu@uc.edu (J.X.); paulca@ucmail.uc.edu (C.P.); shrimp@suda.edu.cn (H.L.); min.liu@uc.edu (M.L.); wanyy@ucmail.uc.edu (Y.-G.W.); MASHRAF@augusta.edu (M.A.)

**Keywords:** Wnt11, mesenchymal stem cells, angiogenesis, ischemic myocardium, Wnt-PKC-JNK pathways

## Abstract

Background: We demonstrated that the transduction of Wnt11 into mesenchymal stem cells (MSCs) (MSC^Wnt11^) promotes these cells differentiation into cardiac phenotypes. In the present study, we investigated the paracrine effects of MSC^Wnt11^ on cardiac function and angiogenesis. Methods and Results: Conditioned medium was collected from MSC^Wnt11^ (CdM^Wnt11^) and their control cells (CdM^GFP^). CdM^Wnt11^, especially obtained from MSC^Wnt11^ exposed to hypoxia, significantly promoted human umbilical vein endothelial cells (HUVECs) migration and increased capillary-like tube (CLT) formation, which was blocked by Wnt11 neutralizing antibody. Wnt11 protein was significantly higher in CdM^Wnt11^ compared to that in CdM^GFP^. Directly treating HUVECs with recombinant Wnt11 protein significantly increased CLT formation, which was abrogated by treating cells with the JNK inhibitor SP600125, as well as the PKC inhibitor Calphostin-C. Moreover, the transfection of Wnt11 to HUVECs (H^Wnt11^) significantly increased CLT formation and HUVEC migration, as well as upregulated p-pan-PKC and p-JNK expression. Injection of CdM^Wnt11^ into the peri-infarct region in a rat acute myocardial infarction (AMI) model significantly improved cardiac function, reduced infarct size, and increased myocardial blood flow and blood vessel density in the ischemic area. Conclusion: Wnt11 released from MSC^Wnt11^ increased angiogenesis and improved cardiac function via non-canonical Wnt-PKC-JNK dependent pathways.

## 1. Introduction

Heart failure due to myocardial infarction (MI) is a major cause of morbidity and mortality worldwide, as adult mammalian heart tissue has a limited capability for self-repair. Stem cell-based therapeutics have been shown to improve cardiac function and attenuate infarct size expansion in both experimental and pre-clinical trials [1,2,3,4]. The regenerative capacity of stem cells is primarily associated with attenuation of pathological ventricular remodeling and induction of neovascularization [4,5,6,7,8,9]. Combining stem cell transplantation with therapeutic gene delivery via genetic modulation of donor cells holds promise for the treatment of ischemic cardiovascular disease [5,8,10].

The Wnts are secreted glycoproteins that comprise a family of nineteen proteins in humans. Wnt signaling regulates a variety of cellular activities, including cell survival, proliferation, migration, and gene expression [11,12,13,14,15,16]. Activation of the Wnt signaling system includes canonical and non-canonical pathways due to the different intracellular events after Wnt-Frizzled binding. The activation of canonical Wnt (e.g., Wnt1, Wnt2a, Wnt3a, and Wnt8a) depends on the nuclear localization of β-catenin and subsequent transcriptional activation of target genes [11]. The activation of non-canonical Wnts (Wnt4, Wnt5, and Wnt11) is β-catenin-independent, and the downstream proteins are not well characterized. They may involve several kinases, including protein kinase C (PKC), calcium/calmodulin-dependent kinase (CaMKII), and Jun N-terminal kinase (JNK) [17,18,19].

Many studies have demonstrated the role of Wnt signaling in regeneration of the ischemic myocardium regeneration [20,21]. Wnt modulation may offer a promising therapeutic strategy towards the restoration of myocardial tissues and an enhancement of cardiac functions following infarction [22]. Upregulation of Wnt signaling via treating cardiomyocytes with N-cadherin antibody or CHIR99021 GSK inhibitor results in a significant increase in matured cardiomyocyte proliferation [23]. In addition, the Wnt signaling pathway may be involved in stem cell fate. It has been reported that Wnt/β-catenin signaling pathway is essential for the self-renewal of a variety of mammalian stem cells [24]. The secreted Frizzled-related protein 2 (sFRP2) improves MSC engraftment through increasing stem cell survival and proliferation [25]. Transplantation of lentivirus-Wnt11-transduced skeletal muscle-derived stem cells (MDSCs) into acutely infarct-injured myocardium showed greater survival and cardiac differentiation [26]. Wnt11 overexpression promotes adipose tissue mesenchymal stem cells (ADSCs) differentiating to the NP cells [27] and promotes bone marrow mononuclear cells or other stem cells differentiation into myocardial phenotype [28,29,30].

In our previous studies, we demonstrated that the transplantation of MSCs overexpressing Wnt11 (MSC^Wnt11^) into ischemic myocardium significantly improved cardiac function and protected cardiomyocytes against ischemic injury [31]. The expression of GATA-4, brain natriuretic peptide, islet-1, and α-actinin was significantly upregulated in MSCs overexpressing Wnt11 [28,31]. It is well known that therapeutic angiogenesis has been recognized as a very important strategy for treating coronary vascular disease. MSC^Wnt11^ can enhance new blood vessel formation [32,33,34]. Many studies, including ours, implicate that MSCs secrete paracrine factors that orchestrate interactions within the cellular milieu to promote angiogenesis [8,32,35,36,37,38,39]. However, it is unclear whether paracrine factors released from MSC^Wnt11^ play an important role in MSC^Wnt11^-mediated heart repair and blood vessel formation. In this study, we aimed to examine the effect of paracrine factors of MSC^Wnt11^ on blood vessel formation and related ischemic myocardial repair and to elucidate the underlying mechanisms.

## 2. Material and Methods

### 2.1. Animals

Sprague–Dawley (SD) rats were obtained from Charles River Laboratories. The Animal Care Committee of the University of Cincinnati Institutional Animal Care and Use Committee approved the experimental protocol. All animal experiments were conducted in accordance with the National Institutes of Health guide for the care and use of laboratory animals (NIH publication No. 85-23, Revised 1996).

### 2.2. Cell Culture and Transduction with Wnt11 Plasmid

Primary cultured MSCs were isolated from femurs and tibias of Sprague–Dawley (SD) rats, as described previously with minor modification [4]. Cells were cultured with Iscove’s Modified Dulbecco’s Medium (IMDM) supplemented with 10% fetal bovine serum (FBS), as well as penicillin (100 U/mL) and streptomycin (100 μg/mL) at 37 °C in humid air with 5% CO_2_. HUVECs were purchased from American Type Culture Collection (ATCC, Manassas, VA, USA) and cultured on the 0.1% gelatin pre-treated plates in endothelial growth medium (Cell Application Inc., San Diego, CA, USA).

Using the murine stem cell virus (pMSCV) retroviral expression system, HUVECs and MSCs were transfected with Wnt11 [28]. Briefly, Wnt11 excised from pcDNA-Wnt11 was cloned into pMSCV-EGFP. pMSCV-EGFP-Wnt11 were then transfected with package vector GP2-293 cells (Clontech, Mountain View, CA, USA) using x-tremeGENE9 DNA transfection reagent (Roche, Indianapolis, IN, USA). After 24 h, supernatant from GP2-293 was collected. HUVECs and MSCs were then incubated with the filtered supernatant for 12 h in the presence of 10 μg/mL polybrene (Sigma, St. Louis, MO, USA). HUVEC and MSC clones with stable expression of Wnt11 were obtained by puromycin (3 μg/mL) (Sigma, St. Louis, MO, USA) selection for 5 days. MSCs and HUVECs negative control were both transduced with pMSCV-IRES-EGFP.

### 2.3. Quantitative Real-Time PCR

Total RNA from MSCs and HUVECs was extracted following manufacturer’s instructions in the RNeasy mini kit (Qiagen, Valencia, CA, USA). Quantitative real-time PCR (RT-PCR) was carried out using an iQ SYBR Super-mix (Bio-Rad, Hercules, CA, USA) on an iQ5 real-time system [28]. In brief, cDNA was synthesized using SuperScript^™^ III First-Strand Synthesis for RT-PCR (Invitrogen, Carlsbad, CA, USA) and amplified using *Taq* DNA polymerase in the presence of primers (Table 1). The expression of target mRNA relative to Glycer-aldehyde-3-phosphate dehydrogenase (GAPDH) was calculated based on the threshold cycle (*C*_T_) as *r* = 2^−Δ (ΔCT)^, where Δ*C*_T_ = *C*_T_ target − *C*_T GAPDH_ and Δ(Δ*C*_T_) = Δ*C*_T experimental_ − Δ*C*_T control_.

### 2.4. Western Blotting

Cells were lysed using lysis buffer (Qproteome Mammalian Protein Prep Kit, Germantown, MD, USA). Protein concentrations of cell lysate were measured with Bio-Rad DC-Protein Assay Reagent (Bio-Rad, Hercules, CA, USA). Proteins were separated using 10% sodium dodecyl sulfate (SDS)–polyacrylamide gel electrophoresis (PAGE) and transferred to a nitrocellulose membrane (Bio-Rad, Hercules, CA, USA). The membrane was blocked with 5% milk Blocking Buffer, and probed overnight at 4 °C with primary antibodies (1:1000) against Wnt11 (R&D, Minneapolis, MN, USA), ERK1, p-p38, p38 (Santa Cruz Biotechnology, Santa Cruz, CA, USA), total-JNK, p-JNK, p-ERK, p-PKC(pan), PKCα, and β-actin (Cell Signaling Technology, Beverly, MA, USA), followed by incubation for 1 h with Horseradish peroxidase (HRP)-conjugated secondary antibody (1:2000) at room temperature. The bends of proteins were detected using an ECL plus kit (GE Healthcare, Pittsburgh, PA, USA). Blot densities were analyzed with NIH image software (AlphaEase FC, version 6.0.0).

### 2.5. Immunocytochemistry

MSCs or HUVECs (2 × 10^4^ cells) were seeded on the 0.1% gelatin pre-treated glass coverslips in 12 well plates and fixed in 4% paraformaldehyde. The fixed cells were blocked in 10% goat serum for 1 h and incubated with 1st antibodies (1:200 dissolved in blocking buffer) against Wnt11, GFP overnight at 4 °C. After being washed, cells were incubated with secondary antibodies of goat anti-mouse IgG or goat anti-rabbit IgG conjugated with Alexa Fluor-568 (Invitrogen, Carlsbad, CA, USA) for 1 h at room temperature. Nuclei were stained with 4′,6-diamino-2-phenylindole (DAPI). Images were taken under an Olympus BX63 microscope (Olympus, Melville, NY, USA) equipped with cellSens Life Science imaging software version 510 (Olympus, Melville, NY, USA).

### 2.6. Collection of Conditioned Medium (CdM)

CdM from cultured MSCs was collected as described in our previous report [35]. Briefly, MSCs were seeded at 5 × 10^6^ in a 15-cm plate for 24 h, and then the culture medium was replaced with serum-free IMDM. In hypoxic treatment, low glucose Dulbecco’s Modified Eagle’s Medium (DMEM) was used and the cells were exposed to hypoxic conditions (1% O_2_, 5% CO_2_, 94% N_2_) (O_2_/CO_2_ incubator-MCO-18M, Sanyo, Japan) for 36 h. The medium was collected and centrifuged (2500 rpm for 3 min) to remove cell debris. The supernatant was then transferred to ultra-filtration conical tubes with membranes selective for <5 kDa (Amicon Ultra-15, MilliporeSigma, Burlington, MA, USA), and centrifuged (4 °C, 3200 g for 45 min) to produce the final CdM at 200 μL/5 × 10^6^ cells.

### 2.7. CLT Formation and Endothelial Cell Migration

Capillary morphogenesis of HUVECs was performed on the Matrigel surface [40]. HUVECs pre-labeled with PKH26 or PKH67 cell tracker (Sigma, St. Louis, MO, USA) were seeded in 24-well plates (3 × 10^4^ cells/well) that were pre-coated with 300 μL of Matrigel Basement Membrane Matrix (BD Biosciences, San Jose, CA, USA). CdM was added into the medium at a ratio of 1:10 fresh medium without serum. Images were automatically taken every hour under IncuCyte Zoom^TM^ live-cell kinetic imaging system (Essen BioScience, Ann Arbor, MI, USA) or manually taken using an Olympus BX 41 microscope. The formation of CLT was quantified by counting tube numbers and calculating the cumulative tube length in five random microscopic fields.

Endothelial cell migration was evaluated by counting sprouts of HUVEC spheroids and directly measuring HUVEC migration. HUVEC spheroids were generated in non-adhesive round-bottom 96-well plates. Each well consisted of 400 cells suspended in EBM-2 medium with 2% FBS and 20% methylcellulose (Sigma, St. Louis, MO, USA) [41]. Spheroids were then embedded in 3D Collagen Cell Culture System (Chemicon, Temecula CA, USA) for 24 h with different treatments, including CdM-fresh medium (1:10) and absence of CdM. Images were taken with an Olympus BX 41 microscope equipped with Olympus digital camera. The number of sprouts from each spheroid was counted and cumulative sprout length was calculated, as described in our previous report [35].

Endothelial cell migration was measured using the BD Biocoat™ Angiogenesis System (BD Biosciences, San Jose, CA, USA) which is composed of a BD Falcon™ 24-Multiwell Insert Plate, and a non-tissue culture (TC)-treated receiver plate. The insert plate was fitted with a fluorescence blocking, microporous (3.0 μm pore size) PET membrane which was evenly coated with Human Fibronectin (Migration System). HUVECs were starved for 5 h prior to setting up the assay. Then cell monolayers were labeled in situ with DilC_12_ (10 μg/mL) [42] in the medium with serum for 1.5 h. Pre-labeled cells were trypsinized and seeded at 1 × 10^5^ cells/250 μL/well in the top insert, while 750 μL culture medium with different CdM was added into the bottom chambers and incubated for 22 h. The numbers of cells that attached to underside of the insert membrane was quantitated as fluorescence intensity using the microplate reader SpectraMax M3 (Molecular Devices, San Jose, CA, USA) read from the bottom at wavelengths of λex/λem = 549 nm/565 nm.

### 2.8. Rat Myocardial Infarction (MI) Model

SD female rats (2- to 3-month-old) were used to create a MI model by permanent ligation of the left anterior descending coronary artery (LAD). Animals were anesthetized with ketamine (100 mg/kg body weight ip) and xylazine (5 mg/kg body weight ip) and then mechanically ventilated. The heart was exposed after a left thoracotomy was performed at the fourth intercostal space using sterile technique. LAD was ligated with a 7-0 Ethicon suture just below the atrioventricular border. Then, hearts received multiple injections of CdM (total 50 μL) or same amount PBS as control, around the border of infarct area using a 27-gauge needle. The chest was then closed and the animal was given buprenorphine (0.5 mg/kg sc) to alleviate pain. In addition, one sham surgery group was included, in which animals underwent chest open without LAD ligation.

### 2.9. Cardiac Function Assessment

An echocardiography study was performed using an HDI 5000 SonoCT (Phillips, St. Cloud, FL, USA) with a 15-MHz probe in rats at 4 weeks post ligation of LAD. Cardiac pump parameters were collected from 2-D images and M-mode interrogation in the long-axis view. LV internal dimensions (LVID) were measured at both diastole (LVIDd) and systole (LVIDs). LV percent fractional shortening (FS) and LV ejection fraction (EF) were calculated as follows: FS = (LVIDd − LVIDs)/LVIDd × 100; EF = [(LVIDd)^3^ − (LVIDs)^3^]/(LVIDd)^3^ × 100. The data were averaged from 3 consecutive cardiac cycles.

### 2.10. Myocardial Blood Flow Measurement and Myocardial Blood Vessel Staining

Following the echocardiography study, myocardial blood flow was measured as described in our previous report [35]. Blue fluorescent microspheres (10-μm) (7.2 × 10^5^ in 200 μL) were injected into the left atrium. A reference blood sample was collected from the descending aorta at a rate of 1 mL/min. The heart was excised, then, the border, infarcted, and normal regions (right ventricle) were dissected and weighted, respectively. Microspheres were extracted from the heart and blood using potassium hydroxide. Blue fluorescence absorbance was measured. Blood flow in border and infarcted areas (Q_s_) was calculated based on Q_s_ (mL/min/g tissue) = (A_s_/A_r_)Q_r_ (mL/min)/W_t_ (g). A_s_ and A_r_ are the absorbance of sample and of reference blood; Q_r_ is the withdrawal rate of the reference blood; W_t_ is tissue weight.

The blood vessel density in myocardium was immunostained with vWF antibody (Santa Cruz Biotechnology, Santa Cruz, CA, USA) (1:200) overnight at 4 °C, and then incubated with secondary antibodies for 1 h at room temperature. Images were taken under an Olympus BX63 microscope equipped with cellSens life science imaging software version 510. A vWF positive cell, single or cluster, was counted as one microvessel. 

The infarction size and LV anterior wall thickening were also determined by staining the sliced heart sections (5 μm thick) with Masson’s trichrome. The images were taken using an Olympus BX41 microscope with a charge-coupled device camera (Olympus, Melville, NY, USA). Left ventricle anterior wall thickening was expressed as a percentage of interventricular septum thickness, and calculated using ImageJ analysis software (version 1.6065; NIH).

### 2.11. Statistical Analysis

The in vitro experiments were carried out in triplicate and repeated at least 3 times. The in vivo data were obtained by an investigator who was blind to the animal groups. Quantitative data were presented as Means ± SEM. Statistical comparisons were performed using ANOVA analysis or Student’s two-tailed paired *t*-test. Differences were considered significant if the *p*-value was less than 0.05.

## 3. Results

### 3.1. Wnt11 Is Upregulated in MSC^Wnt11^

**Figure 1:**
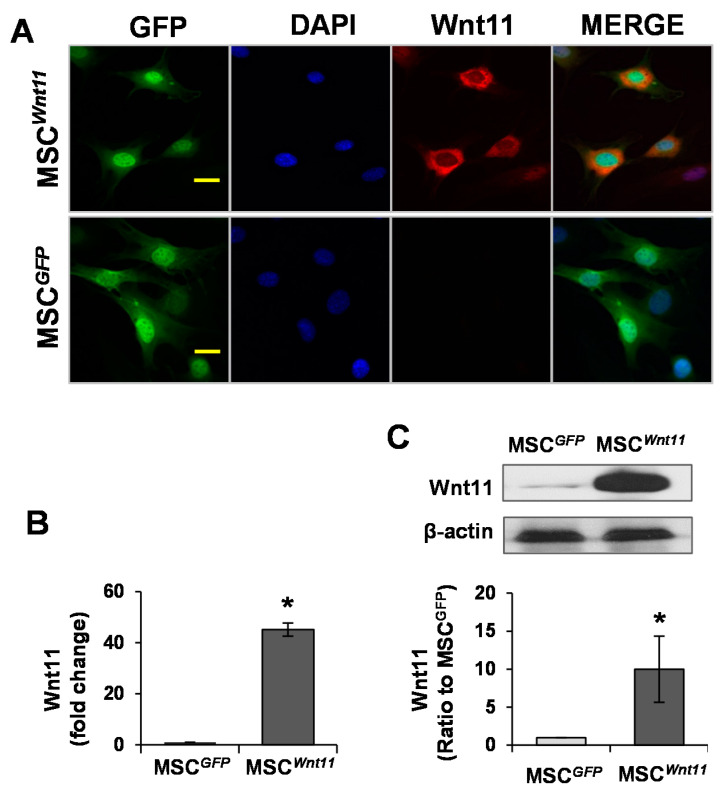
Wnt11 is upregulated in MSC^Wnt11^. (**A**): Mesenchymal stem cells (MSCs) were transduced with Wnt11 using the murine stem cell virus (pMSCV) retroviral expression system and then subjected to immunostaining with Wnt11 (Red) and GFP (Green). Nuclei were counterstained with DAPI (blue). The scale bars represent 50 μm. (**B**): RT-PCR showed that the mRNA of Wnt11 is significantly increased in MSC^Wnt11^. (**C**): Western blot showed the relative expression of Wnt11 normalized to GAPDH in each sample and compared with the levels in MSC^GFP^. *, *p* < 0.05 vs. MSC^GFP^.

The expression of Wnt11 in MSCs was evaluated using immunostaining, real-time PCR, and Western blotting. MSC^Wnt11^ were intensely positive for Wnt11 staining (Figure 1A), and the expression of Wnt11 in MSC^Wnt11^ at mRNA level was 40-fold higher than that in MSC^GFP^ (Figure 1B). Western blotting results confirmed the elevated Wnt11 expression at the protein level in MSC^Wnt11^ in comparison with MSC^GFP^ (Figure 1C).

### 3.2. CdM^Wnt11^ Promoted Capillary-Like Tube (CLT) Formation and Sprouting of HUVEC Spheroids

**Figure 2:**
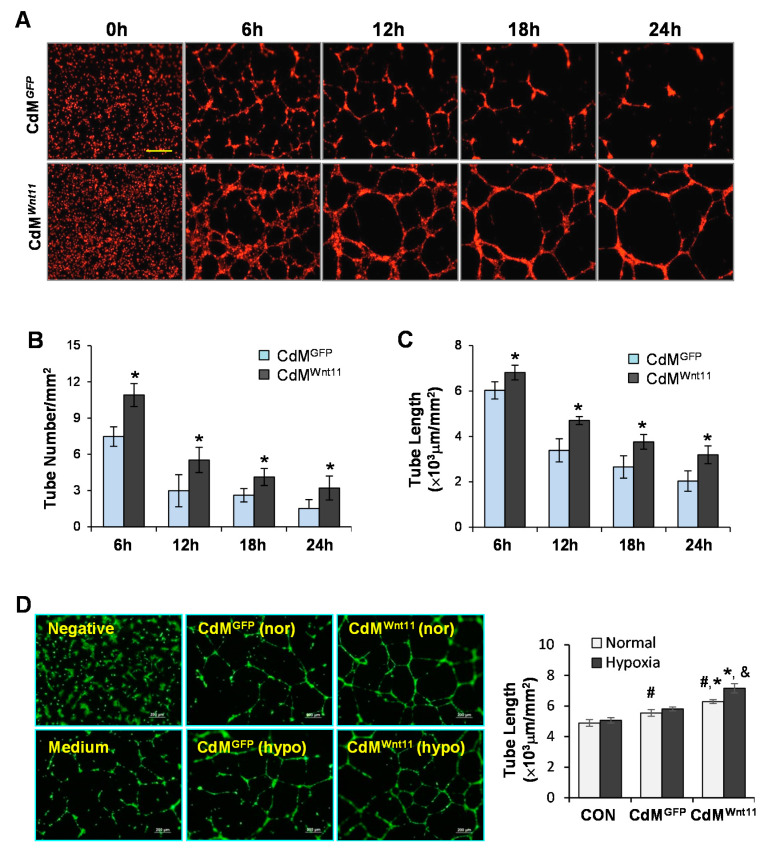
CdM^Wnt11^ significantly promote capillary-like tube (CLT) formation. (**A**): Representative real-time images of CLT formation following human umbilical vein endothelial cell (HUVEC) treatment with CdM^Wnt11^ and CdM^GFP^. The scale bars represent 800 μm. (**B**,**C**): Quantitative analysis of the tube number and length. (**D**): CLT formation of HUVECs treated with various CdM collected from MSCs exposed to normal or hypoxia. ^#^, *p* < 0.05 vs. CON; *, *p* < 0.05 vs. CdM^GFP^; ^&^, *p* < 0.05 vs. CdM obtained from MSCs exposed to normal culture, respectively.

**Figure 3:**
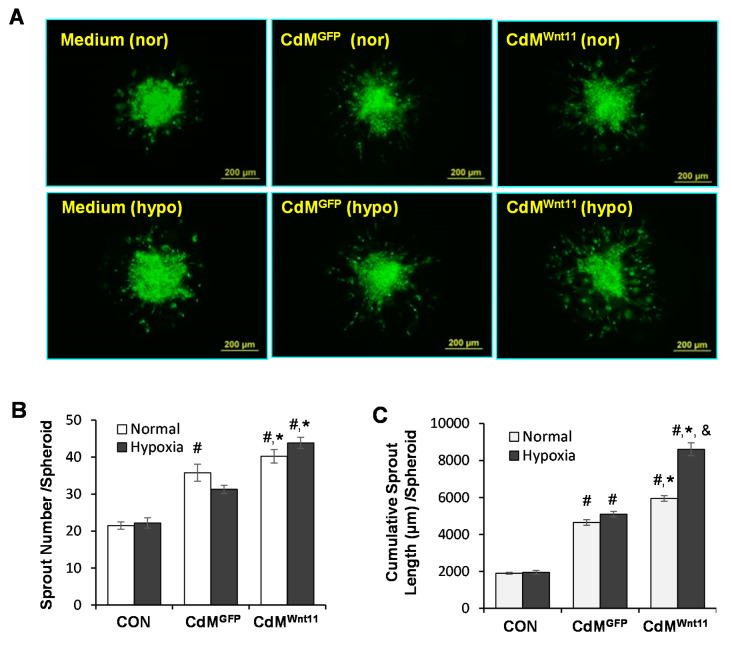
CdM from MSCs overexpressing Wnt11 promotes angiogenic sprouting of HUVECs. (**A**): Representative images of HUVEC sprout from spheroids underwent different treatments for 24 h. (**B**): Quantitative data of sprout number from each spheroid. (**C**): Quantitative data of sprout length from each spheroid. ^#^, *p* < 0.05 vs. CON; *, *p* < 0.05 vs. CdM^GFP^; ^&^, *p* < 0.05 vs. CdM obtained from MSCs exposed to normal culture, respectively.

Real-time recorded images of CLT formation showed that small tubes were formed at the beginning. Tube size then increased and tube number reduced with the time extension during the recording period. Treatment of HUVECs with CdM^Wnt11^ not only significantly increased CLT length and number at the beginning, but also maintained the CLT longer when compared to HUVECs treated with CdM^GFP^ (Figure 2A–C). Moreover, the number of CLT was significantly higher in HUVECs treated with CdM collected from MSC^Wnt11^ that were exposed to hypoxia compared to CdM obtained from MSC^Wnt11^ under normal culture (Figure 2D). The number of sprouts and the cumulative sprout length were both higher in CdM^Wnt11^-treated HUVECs, especially in CdM obtained from MSC^Wnt11^ exposed to hypoxia (Figure 3). These results indicate that CdM^Wnt11^, especially the CdM collected from MSC^Wnt11^ that were exposed to hypoxia, increases CLT formation and promotes endothelial cell migration.

### 3.3. Wnt11 Plays an Important Role in CdM^Wnt11^-Mediated Angiogenesis

**Figure 4:**
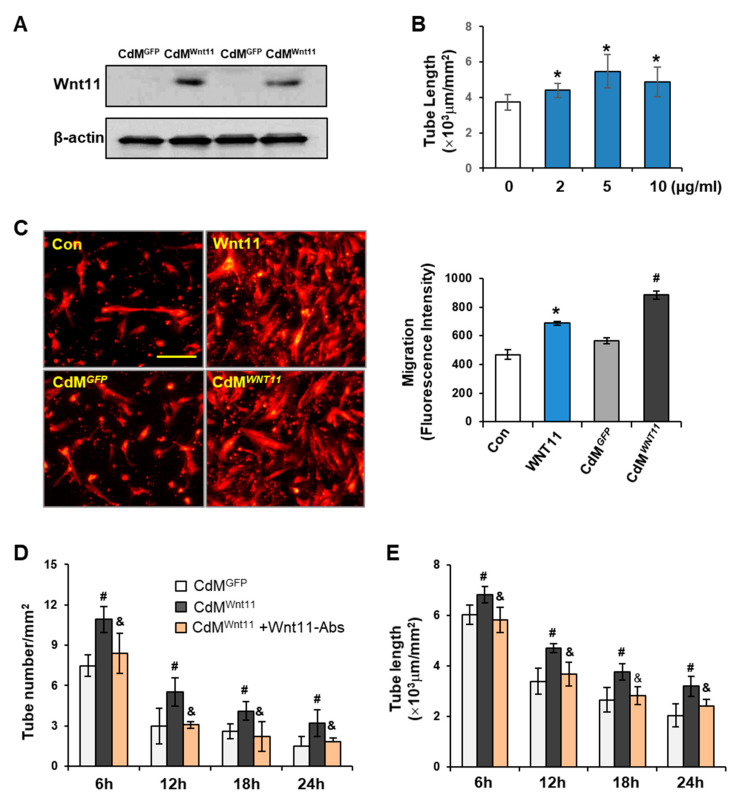
Wnt11 promotes CdM^Wnt11^-mediated capillary-like tube (CLT) formation and HUVEC migration. (**A**): Representative images of Western blot on expression of Wnt11 in CdM^Wnt11^ and CdM^GFP^. (**B**): The effect of Wnt11 on HUVEC-mediated CLT formation. (**C**): Representative images of HUVEC migration and quantitative data of fluorescence intensity. The scale bars represent 100 μm. (**D**,**E**): Wnt11 neutralized antibody partially abolished CdM^Wnt11^-mediated CLT formation. *, *p* < 0.05 vs. Control, ^#^ *p* < 0.05 vs. CdM*^GFP^*; ^&^, *p* < 0.05 vs. CdM^Wnt11^, respectively.

Wnt11 protein was significantly increased in CdM^Wnt11^ in comparison to that in CdM^GFP^ (Figure 4A). Addition of Wnt11 (2–10 μg/mL) significantly increased CLT length (Figure 4B). The effect of CdM^Wnt11^ on endothelial cell migration was also tested by seeding HUVECs in BD BioCoat™ Endothelial Cell migration system for 22 h. HUVEC migration was significantly increased in the set that contained CdM^Wnt11^ versus without CdM^Wnt11^. The addition of 5 μg/mL purified Wnt11 alone also significantly increased HUVEC migration (Figure 4C). To directly verify the role of Wnt11 in CdM^Wnt11^-mediated angiogenesis, Wnt11 neutralizing antibody was simultaneously added with CdM^Wnt11^ in HUVEC culture. Wnt11 neutralizing antibody abolished CdM^Wnt11^-mediated CLT formation following culture with HUVECs (Figure 4D,E). 

**Figure 5:**
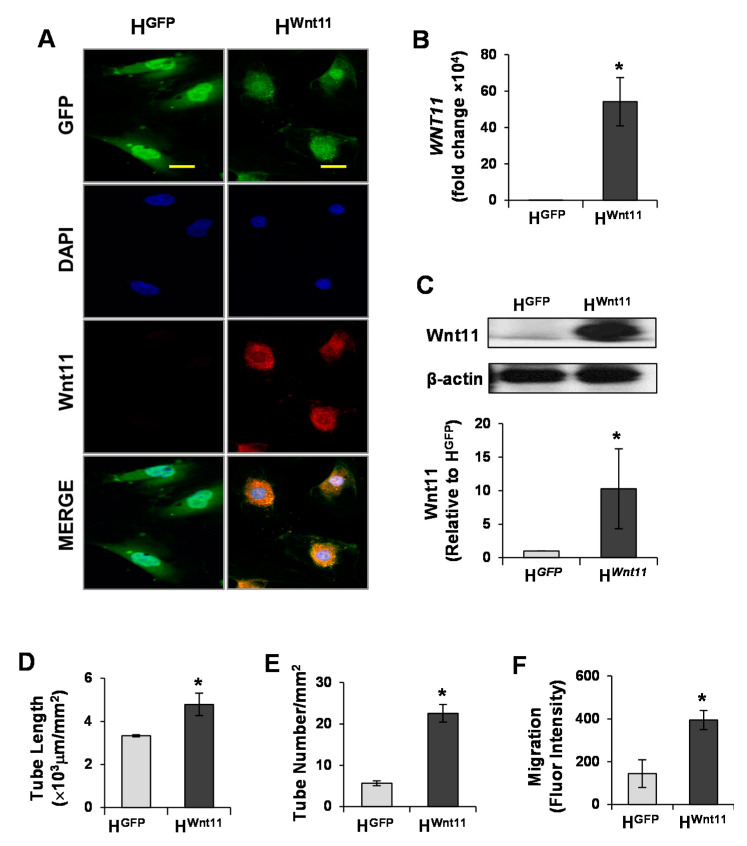
Wnt11 promotes capillary-like tube (CLT) formation and endothelial cell migration. (**A**): HUVECs were directly transduced with Wnt11 using retroviral-mediated *Wnt11/GFP* bicistronic construct and then subjected to immunostaining with Wnt11 (Red) and GFP (Green). Nuclei were counterstained with DAPI (blue). The scale bars represent 50 μm. (**B**): RT-PCR showed that the mRNA of Wnt11 was significantly increased in HUVECs transduced with Wnt11 (H^Wnt11^). (**C**): Western blot showed the relative expression of Wnt11 normalized to β-actin in each sample and compared with the levels in HUVECs transduced with GFP (H*^GFP^*). (**D**,**E**): CLT formation of H*^GFP^* and H*^Wnt11^*. (**F**): HUVEC migration. *, *p* < 0.05 vs. H^GFP^.

To further investigate the direct action of Wnt11 on endothelial cells to promote CLT formation, gain-of-function experiments were performed by overexpressing Wnt11 in HUVECs. HUVECs with stable expression of Wnt11 (H^Wnt11^) were established and cells with stable expression of GFP (H^GFP^) served as a control. Overexpression of Wnt11 at both the mRNA and protein level was shown in H^Wnt11^ cells (Figure 5A–C). The length of CLS in H^Wnt11^ (4.79 ± 0.51 × 10^3^ μm/mm^2^) was significantly longer than that in H^GFP^ (3.33 ± 0.05 × 10^3^ μm/mm^2^) following culture for 16 h (Figure 5D). Similarly, tube number was increased (22.60 ± 2.13/mm^2^), compared with that of H^GFP^ (5.67 ± 0.60/mm^2^) (Figure 5E). Likewise, the migration of H^Wnt11^ was much faster than that of H^GFP^ as measured by fluorescence intensity (Figure 5F).

### 3.4. Wnt11 Activates Non-Canonical PKC/JNK Signaling Pathway

**Figure 6:**
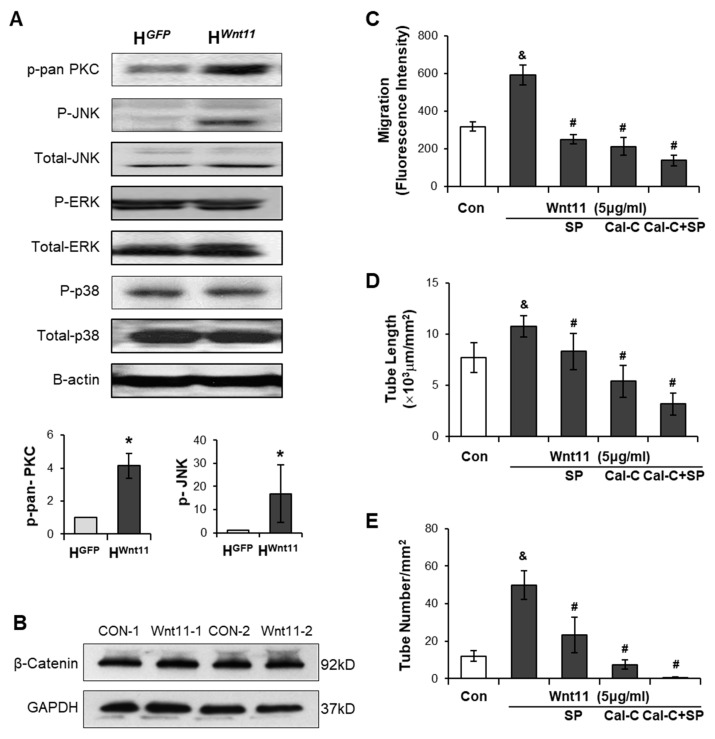
Wnt11 increases HUVEC migration and capillary-like tube (CLT) formations via activating PKC-JNK signaling pathway. (**A**): The protein levels of PKC/JNK signaling pathways in HUVECs were evaluated using Western blot and the relative protein density of p-pan-PKC and p-JNK in H^Wnt11^, compared to those in H^GFP^. (**B**): β-catenin levels in HUVECs treated with/without Wnt11 (5 µg/mL) for 24 h. (**C**): CLT length; (**D**): tube number; and (**E**): HUVEC migration was calculated under different treatment conditions. *, *p* < 0.05 vs. H^GFP^; ^&^, *p* < 0.05 vs. Control; ^#^, *p* < 0.05 vs. Wnt11, respectively.

To investigate whether Wnt11-mediated angiogenesis via activating the non-canonical signaling pathway in endothelial cells, PKC, JNK, ERK, and p38 in HUVECs were evaluated in HUVECs using Western blot. No significant differences in the protein levels of p-ERK and p-38 and total JNK, ERK, were found between H^Wnt11^ and H^GFP^. However, the expression p-pan-PKC and p-JNK was significantly upregulated in H^Wnt11^ when compared to those in H^GFP^ (Figure 6A). We examined the role of Wnt11 on the expression of β-catenin in HUVECs. There was no significant difference in β-catenin levels in HUVECs treated with Wnt11 (5 µg/mL) compared to those treated with PBS (as control) (Figure 6B). To demonstrate if the activations of PKC/JNK pathways were involved in Wnt11-mediated angiogenesis, PKC inhibitor calphostin-C and/or JNK inhibitor SP600125 was introduced into the HUVEC culture. The results showed that Wnt11 (5 μg/mL)-mediated CLT formation and cell migration were completely blocked by Calphostin-C (0.1 μM). However, SP600125 (5 μM) only partially abolished the Wnt11-induced CLT formation. PKC-JNK inhibitors not only significantly reduced Wnt11-mediated cell migration, but also significantly inhibited the basal HUVEC-mediated CLT formation and migration of HUVECs (Figure 6C–E). These data indicated that non-canonical Wnt11 signaling in angiogenesis was dependent on PKC and JNK activation.

### 3.5. CdM^Wnt11^ Improved Cardiac Function via Increasing Regional Blood Flow and Blood Vessel Density

**Figure 7:**
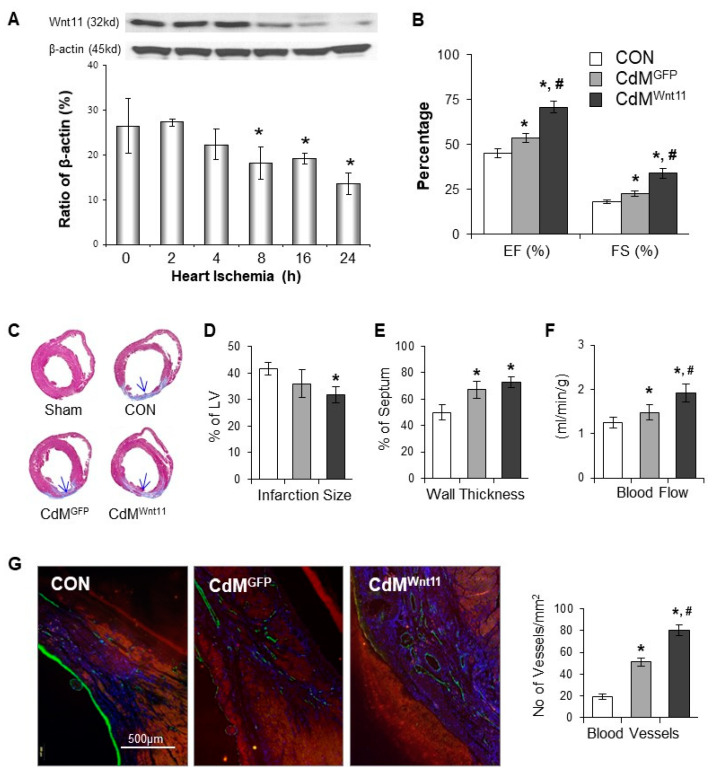
CdM^Wnt11^ significantly improves cardiac contractile function and increases angiogenesis in ischemic myocardium. (**A**): The protein levels of Wnt11 in myocardium following left anterior descending coronary artery (LAD) ligation were evaluated using Western blot and semi-quantitative data. (**B**): Cardiac function calculated from echocardiography images of rats undergone LAD ligation for 4 weeks. (**C**): Representative middle heart sections that were stained with Masson’s trichrome. (**D**,**E**): Quantitative data of left ventricle wall thickness and fibrosis. (**F**): Blood flow in the infarcted and border zones. (**G**): Representative photomicrographs showed vWF-positive cells and the quantitation of vWF-positive capillary density. *, *p* < 0.05 vs. control (CON); ^#^, *p* < 0.05 vs. CdM^GFP^, respectively. In Panel (**A**): *n* = 3; In Panels (**B**,**F**): CON: *n* = 6; CdM^GFP^: *n* = 6, and CdM^Wnt11^: *n* = 7. In Panels (**C**–**E**,**G**), CON: *n* = 6; CdM^GFP^: *n* = 7; and CdM^Wnt11^: *n* = 9.

We explored the expression of Wnt11, Wnt 3 and Wnt5a in ischemic heart tissues at different time points post LAD ligation. Wnt11 protein was significantly reduced in the myocardium after the heart underwent ischemia for 8 h in a time-dependent manner (Figure 7A). However, no significant change was observed in the expression of Wnt3 and Wnt5a. The cardiac function was measured using echocardiography at Week 4 after the CdM was injected into the ischemic myocardium. EF and FS were reduced in LAD-ligated animals, which was significantly improved in animals treated with CdM^Wnt11^ (Figure 7B). Masson trichrome staining showed that LAD ligation induced myocardial infarction. The infarct size was significantly reduced and LV anterior wall thickness was increased in animals treated with CdM^Wnt11^, compared to the animals in the control group, although there was no significant difference compared with the group treated with CdM^GFP^ (Figure 7C–E). The regional blood flow to the infarcted and peri-infarcted areas was very low, which was significantly increased in the animals treated with CdM^Wnt11^ (Figure 7F). The number of microvessels was significantly increased in the rats administrated CdM^Wnt11^ compared to those treated with CdM^GFP^ (Figure 7G).

## 4. Discussion

Our studies demonstrated that conditioned medium collected from MSCs transduced with Wnt11 (CdM^Wnt11^) stimulated endothelial cells to generate capillary-like tube (CLT) and promoted endothelial cell migration. Injection of CdM^Wnt11^ promoted ischemic myocardium regeneration, which is partially mediated by promoting blood vessel formation. The angiogenetic effects of CdM^Wnt11^ might be associated with its high concentrations of Wnt11, which directly activates the Wnt11/non-canonical signaling pathway.

In the present study, we first tracked CLT formation using the IncuCyte Zoom^TM^ live-cell kinetic imaging system, which allowed us to kinetically monitor the angiogenesis process. It was found that CdM^Wnt11^ not only stimulated HUVECs generating CLT earlier, but also kept the formed CLT lasting longer than those treated with CdM obtained from MSCs transduced with control GFP protein (CdM^GFP^). Moreover, CdM^Wnt11^ also promoted endothelial cell migration. We then compared the effect of CdM obtained from MSCs cultured in hypoxic conditions with those collected from MSCs cultured in normal conditions. Interestingly, CdM^Wnt11^ obtained from MSCs exposed to hypoxia significantly promoted CLT formation and HUVEC spheroid sprouting, compared to those obtained from MSCs cultured in normal condition, which might be due to the secretion of more Wnt11 or hypoxia-inducible other factors from MSCs in hypoxic condition. All of these results indicated that CdM^Wnt11^ significantly improved endothelial cell-mediated angiogenesis.

It has been reported previously that the canonical Wnt signaling is activated in the heart following MI [43,44]. We found that the expression of Wnt11 in ischemic myocardium was significantly decreased in a time-dependent manner. Intramuscular injection of CdM^Wnt11^ in a rat MI model markedly reduced infarct size and improved cardiac function with a significant increase in capillary numbers and blood flow in the ischemic area. We measured the Wnt11 in CdM^Wnt11^ with semi-quantitative Western blotting. The concentration of Wnt11 in CdM^Wnt11^ was significantly higher than that in CdM^GFP^, which is consistent with previous reports that Wnt can be secreted from stem cells [45,46]. Therefore, the cardiac injury following ischemia may be, at least partially, due to the loss of non-canonical Wnt signaling. Injection of CdM^Wnt11^ may increase Wnt11, resulting in promoting myocardium regeneration via protecting cardiomyocytes and improving angiogenesis. In this study, we focused on the effect of CdM^Wnt11^ on angiogenesis, but did not include a group in which purified Wnt11 was directly injected into the border of ischemic myocardium. To further elucidate the therapeutic effect of Wnt11-mediated myocardial regeneration, we are planning to inject purified Wnt11 directly into the ischemic myocardium in future studies.

The role of Wnt11 in CdM^Wnt11^-mediated blood vessel formation was examined by inhibiting Wnt11 signaling using Wnt11 neutralizing antibody, and by treating HUVECs with recombinant Wnt11 protein or by directly transfecting Wnt11 into HUVECs. We show that disruption of Wnt11 signaling using the Wnt11 neutralizing antibody led to the less of endothelial cell migration and the ability to form capillary like tubes. In contrast, the overexpression of Wnt11 in HUVECs or adding recombinant Wnt11 protein to the HUVECs angiogenic system had more capillary-like tube formation and cell migration. These results suggest that Wnt11 released from MSC^Wnt11^ plays an important role in CdM^Wnt11^-mediated angiogenesis. Our findings are consistent with previous reports that non-canonical Wnts are involved in angiogenesis by increasing endothelial cell proliferation and cell survival [47,48,49]. In Figure 4C, the migration of HUVEC is stimulated considerably more with the CdM^Wnt11^ than that with purified Wnt11. This effect implied that either an increased concentration of Wnt11 in CdM^Wnt11^ or increased secretion of growth factors as well as other angiogenetic stimulators from MSCs transduced with Wnt11. These factors can interact with Wnt11 to increase migration of HUVECs. However, the different results on angiogenesis following treatment with Wnt11 or non-canonical Wnt signaling have also been reported, e.g., using AAV9-mediated overexpression of Wnt11, Morishita and colleagues [20] showed an anti-inflammatory effect in a mouse model of MI, without any effect on angiogenesis. Therefore, more experiments are needed to elucidate the detailed mechanisms of how Wnt11 regulates angiogenesis.

Wnt11 is considered to be most often associated with non-canonical Wnt signaling [50]. Targeting non-canonical Wnt pathways has led to recent exciting advances in angiogenesis including tumorigenesis, vascular development, endothelial cell proliferation and migration [47,51]. However, the downstream of Wnt11 signaling in endothelial cell biology and neovascularization is not well characterized. Wnt11 triggers intracellular Ca^2+^ release, which then activates Ca^2+^-sensitive kinases, e.g., protein kinase C (PKC) and calmodulin-dependent protein kinase II (CamKII). We then examined the expression of CamKII and PKC in HUVECs and found that the protein level of CamKII and phospho-pan-PKC was upregulated in HUVECs transduced with Wnt11. In addition, inhibition of PKC with calphostin-C markedly reduced Wnt11-mediated CLT formation and HUVEC migration. These results suggest that Wnt11-mediated angiogenic response is dependent on the activation of the PKC pathway. A previous study also demonstrated that PKC inhibitors inhibited the activation of JNK, suggesting that PKC is upstream of JNK [30,52]. In our studies, JNK was also upregulated in HUVECs transduced with Wnt11. Chemical inhibition of JNK using SP600125 could efficiently block the Wnt11-mediated angiogenic effect. Taken together, a greater redundancy for Wnt-PKC-JNK signaling is involved in CdM^Wnt11^-mediated angiogenesis. Our results are supported by several previous studies showing that activated PKC is involved in Wnt-mediated angiogenesis [53] and PKC is upstream of JNK [30,52].

Although we did not find any effect of Wnt11 on the expression of β-catenin in endothelial cells, it has been reported that non-canonical Wnt11 signals may inhibit Wnt canonical activity via preventing stabilization of the β-catenin protein [54]. Pharmacologic inhibition of Wnt canonical (β-catinin-dependent) pathway significantly reduced the decline in cardiac function, prevented adverse cardiac remodeling, and reduced infarct size [43]. Wnt10b is expected to activate the Wnt/β-catenin pathway. The overexpression of Wnt10b was shown to induce robust neovascularization by Paik et al. [44]. In contrast, Wnt antagonist, Dickkopf2, promoted angiogenesis through inhibiting β-catenin signaling pathways [55]. Therefore, it is also possible that Wnt11 in CdM^Wnt11^ may indirectly inhibit β-catenin-dependent canonical Wnt signaling to promote angiogenesis. The role of canonical Wnt signaling in CdM^Wnt11^-mediated angiogenesis should be examined in the future.

In conclusion, Wnt11 released from MSC^Wnt11^ improved cardiac function recovery from acute ischemic injury by promoting endothelial cell-mediated angiogenesis through activation of the non-canonical Wnt-PKC-JNK pathways. Our studies suggest that Wnt signaling network has the potential to play an important role in the regulation of endothelial cell fate in vascular remodeling.

## Figures and Tables

**Table 1:** Primers used in this study.

Primers	Forward	Reverse
Wnt11	5′-CAGGATCCCAAGCCAATAAA	5′-GACAGGTAGCGGGTCTTGAG
GFP	5′-AAGTTCATCTGCACCACCG	5′-TCCTTGAAGAAGATGGTGCG
GAPDH	5′-GTATGACAACGAATTTGGCTACAG	5′-TGAGGGTCTCTCTTCCTCTTGT
